# Supplementary material for: Editor’s Choice Platform for identifying human glycan-specific antibodies against bacterial pathogens using synthetic glycan fragments
Source: Glycobiology. 2025 Oct 10;35(11):cwaf064. doi: 10.1093/glycob/cwaf064 (PMC12596258; doi:10.1093/glycob/cwaf064)
Supplement: Supplementary_data_cwaf064 [file supplementary_data_cwaf064.pdf]

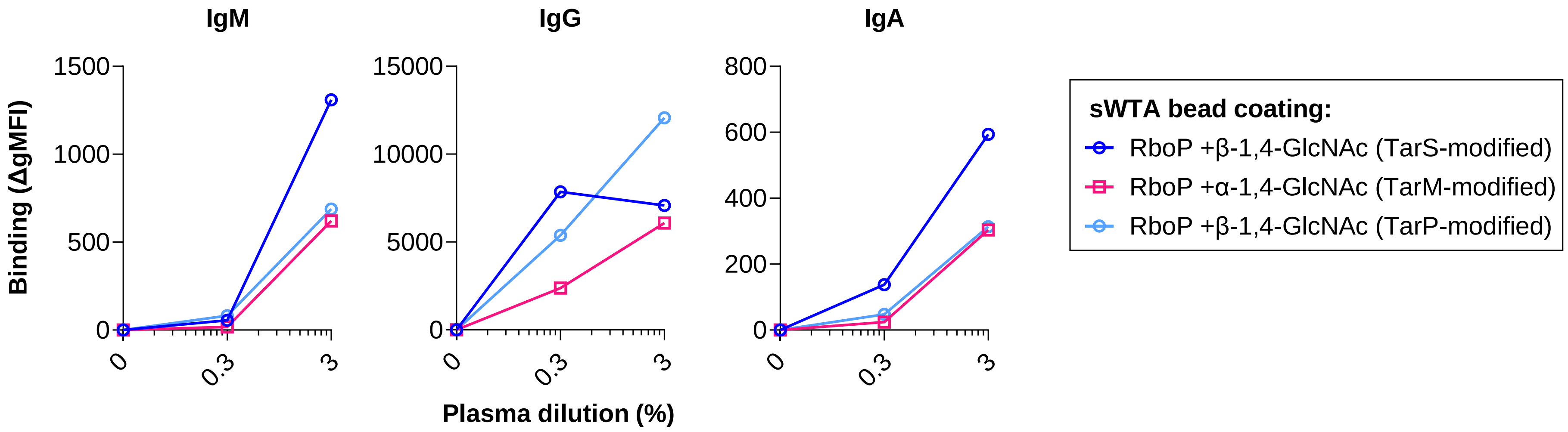

Figure S1: Profiling healthy donor buffy coat plasma for sWTA-specific antibodies

Isotype profiling of healthy donor (HD) plasma for sWTA reactivity. Plasma was titrated and incubated with beads coated with RboP +β-1,4-GlcNAc, +α-1,4-GlcNAc, or +β-1,3-GlcNAc. Bead-bound IgG, IgM, and IgA were measured using flow cytometry. Data represent gMFI values after subtraction of the gMFI value belonging to 0% dilution to remove background variation (i.e. ΔgMFI = gMFI mAb − gMFI no mAb).

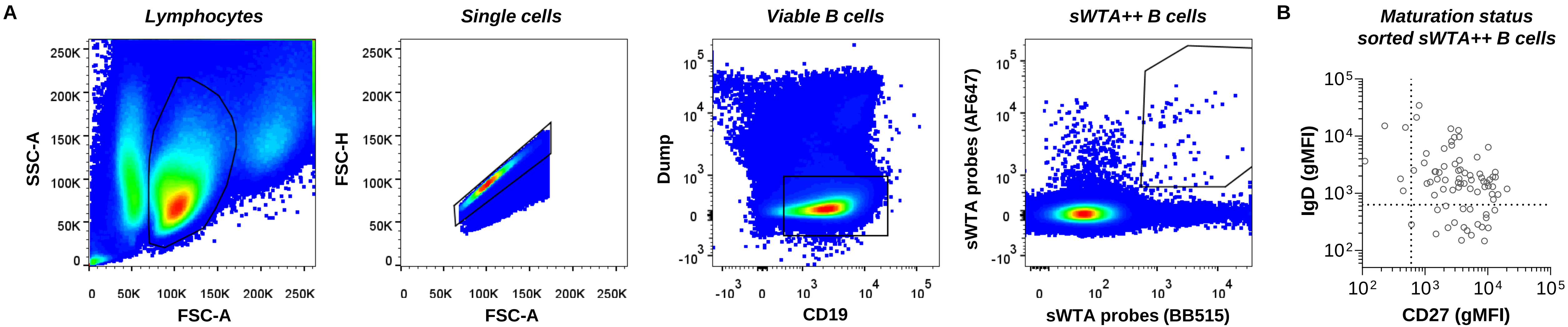

Figure S2: Gating strategy (A) and maturation status (B) of sorted sWTA probe-labelled B cells
Cut off values for maturation marker expression are indicated by dotted lines in order to distinguish unswitched (IgD+ CD27+) and switched (IgD− CD27+) memory B cells, and naïve B cells (IgD+ CD27−).

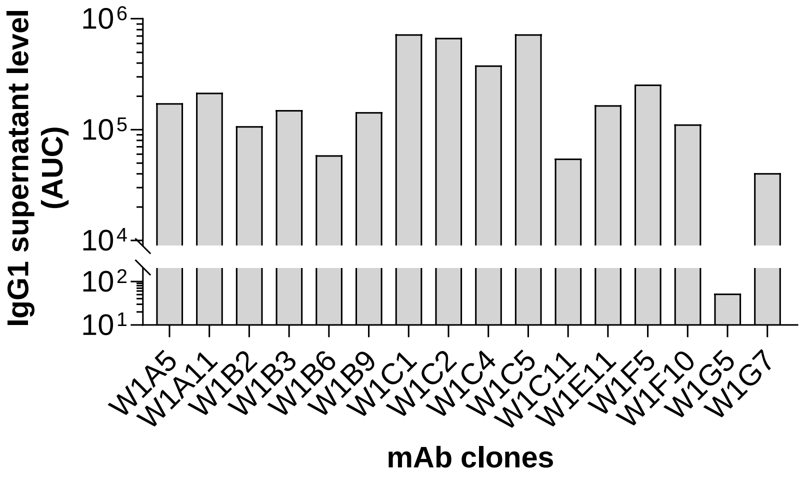

Figure S3: IgG1 levels in pilot-scale productions

Human IgG1 levels in HEK293T cell supernatants of 16 B cell-derived mAbs. Supernatant dilutions were incubated with magnetic protein A-coated beads to determine mAb production levels as measured by flow cytometry. Data represent area under the curve (AUC) values of titration curves ranging from 50% to 6.25% supernatant dilutions. W1G5 was the only clone that exhibited negligible production and was therefore excluded from the specificity screening in main Figure 2.

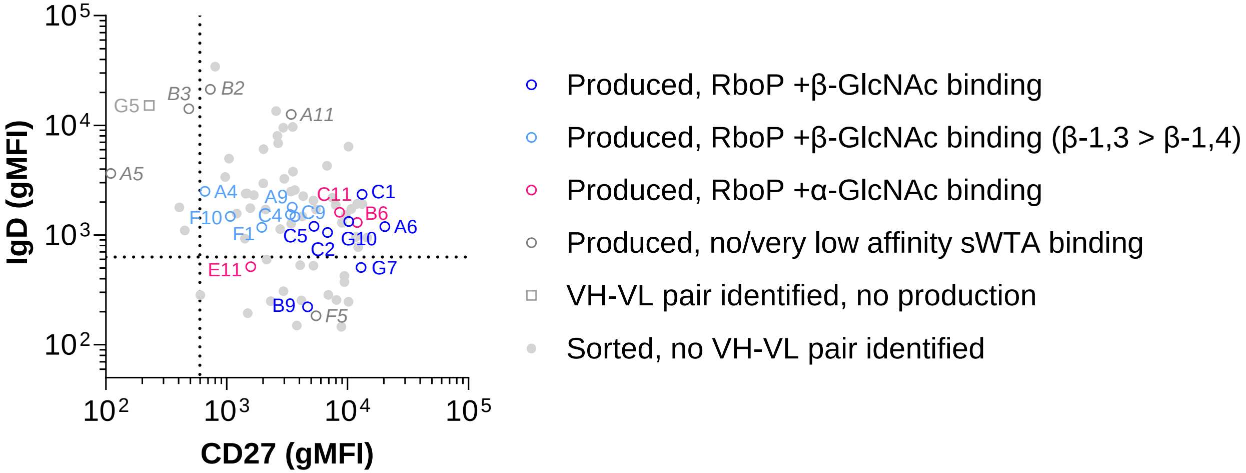

Figure S4: Index sort of sWTA probe-reactive sorted B cells
Maturation status of all sorted B cells. Cells are categorized based on whether the BCR VH-VL pair was identified (or not) for mAb production. The clones with matching VH-VL pairs were further subdivided into those that could be produced (or not) and their reactivity towards sWTA beads as depicted in main Figure 2. Cut off values for maturation marker expression are indicated by dotted lines in order to identify unswitched (IgD+ CD27+) and switched (IgD− CD27+) memory B cells and naïve B cells (IgD+ CD27−).

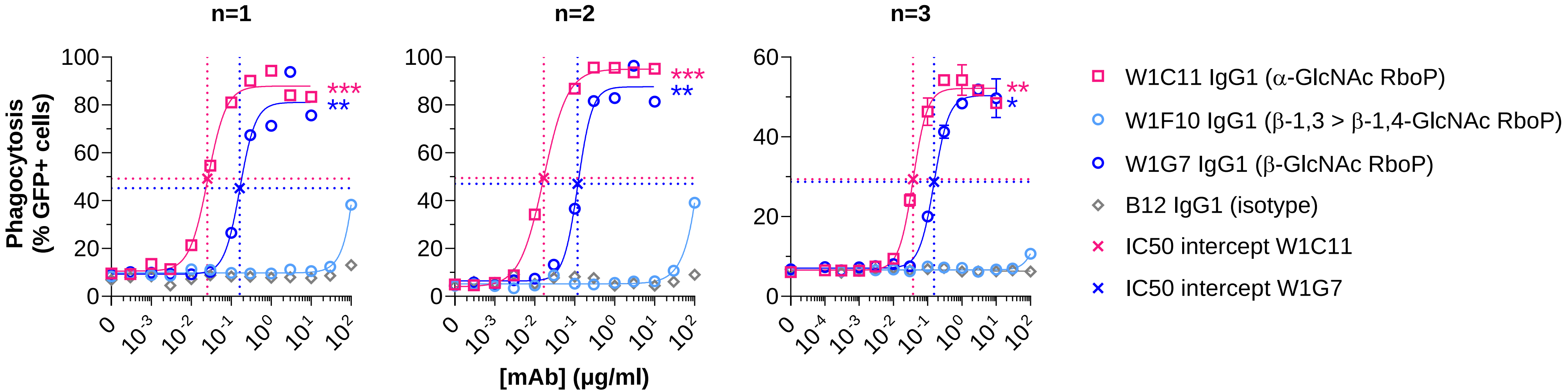

Figure S5: Neutrophil-mediated phagocytosis of GFP-expressing *S. aureus* Newman *Δspa Δsbi* by anti-sWTA mAbs
Each panel represents an individual biological replicate. Reactions were performed in the presence of 1% IgG-/IgM-depleted human serum. Displayed data represent percentages of GFP-positive neutrophils. Curves were generated using nonlinear dose-response fitting model and used to determine the IC_{50} values depicted in main Figure 5 E. Horizontal dotted lines indicate the y-value of 50% effect ([[curve top − bottom] ÷ 2] + bottom) and the mAb concentration of the interception point (cross) with the curve corresponds to IC_{50} (intercept of vertical dotted line with x-axis).

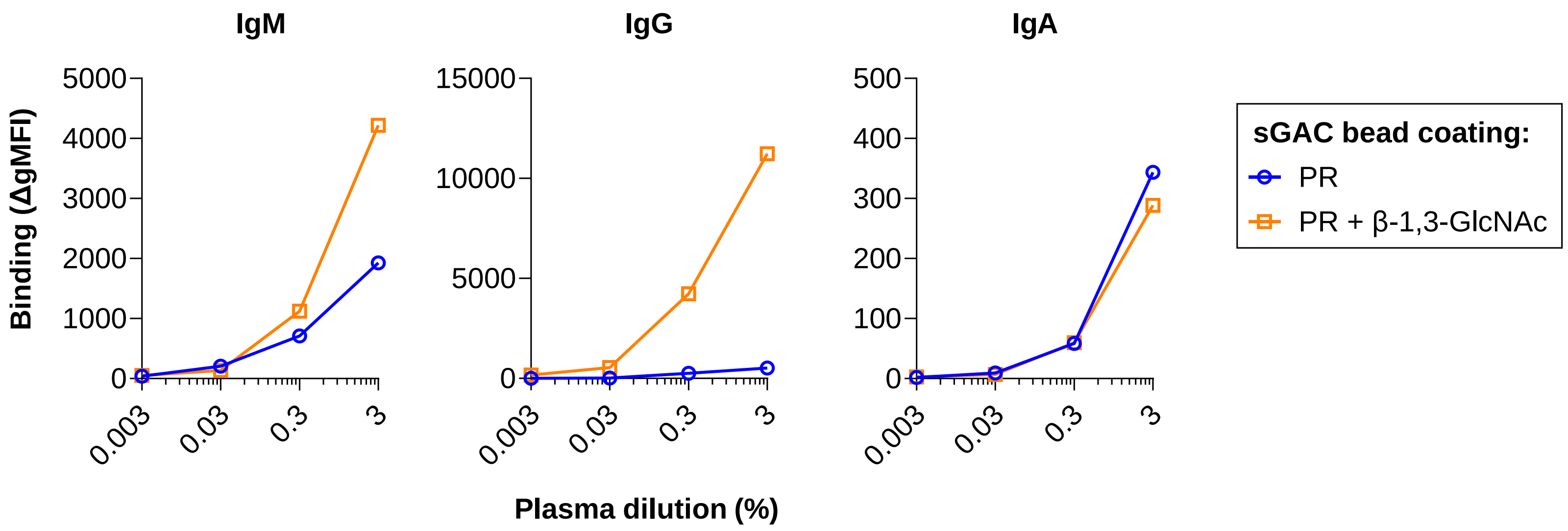

Figure S6: sGAC-specific antibody profiling healthy donor plasma
Isotype profiling of HD plasma for sGAC reactivity. Plasma was titrated and incubated with beads coated with PR or PR +β-1,3-GlcNAc sGAC glycoforms. Bead-bound IgG, IgM, and IgA were measured using flow cytometry. Data represent gMFI values after subtraction of the gMFI value belonging to 0% dilution (i.e. ΔgMFI = gMFI mAb − gMFI no mAb) to remove background variation.

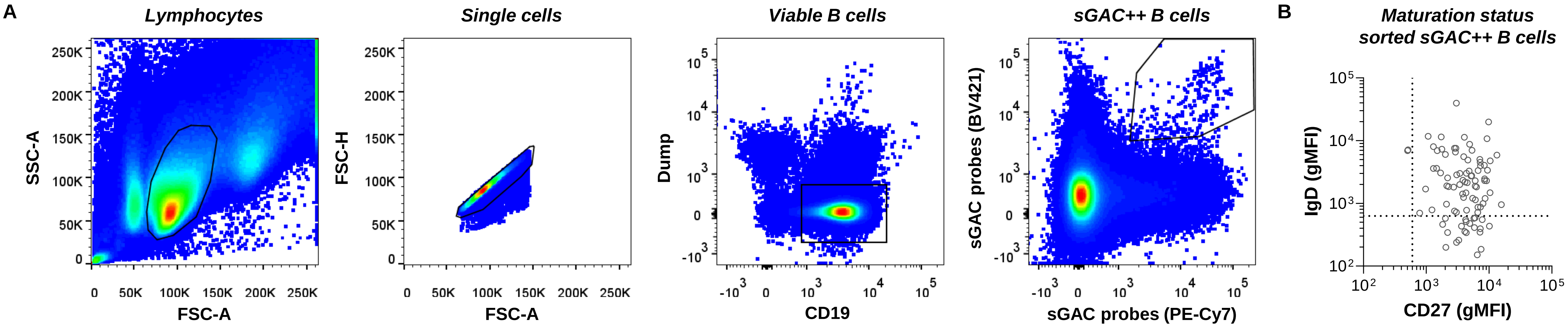

Figure S7: Gating strategy (A) and maturation status (B) of sorted sGAC probe-labelled B cells
Cut off values for maturation marker expression are indicated by dotted lines in order to distinguish unswitched (IgD+ CD27+) and switched (IgD− CD27+) memory B cells.

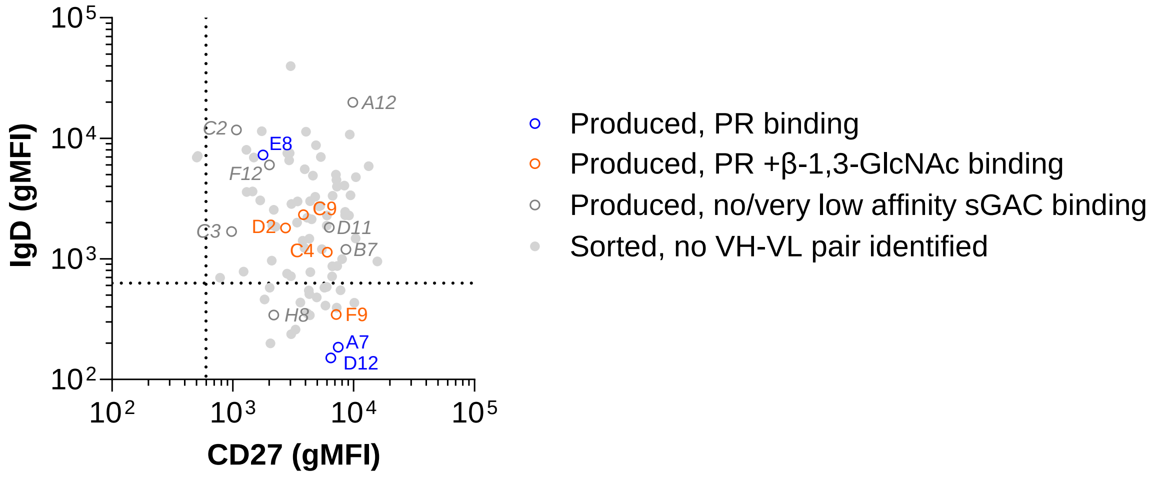

Figure S8: Index sort of sGAC probe-labelled sorted B cells

Maturation status of all sorted B cells. Cells are categorized based on whether the BCR VH-VL pair was identified (or not) for mAb production. The clones with matching VH-VL pairs were further subdivided into those that could be produced (or not) and their confirmed reactivity towards sGAC beads as depicted in main Figure 6 C. Cut off values for maturation marker expression are indicated by dotted lines in order to identify unswitched (IgD+ CD27+) and switched (IgD− CD27+) memory B cells and naïve B cells (IgD+ CD27−).

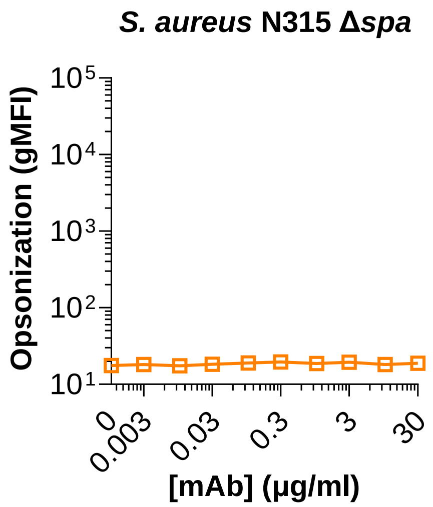

Figure S9: sGAC ß-1,3-GlcNAc-reactive mAb G1C4 is not cross-reactive to ß-1,3-GlcNAc on RboP-based *S. aureus* WTA
